# Possible Ballast Water Transfer of Lionfish to the Eastern Pacific Ocean

**DOI:** 10.1371/journal.pone.0165584

**Published:** 2016-11-02

**Authors:** Hugh J. MacIsaac, Emma M. De Roy, Brian Leung, Alice Grgicak-Mannion, Gregory M. Ruiz

**Affiliations:** 1 Great Lakes Institute for Environmental Research, University of Windsor, Windsor, Ontario, Canada; 2 Department of Biology, McGill University, Montreal, Quebec, Canada; 3 Smithsonian Environmental Research Center, Edgewater, Maryland, United States of America; CSIR-National Institute of Oceanography, INDIA

## Abstract

The Indo-Pacific Red Lionfish was first reported off the Florida coast in 1985, following which it has spread across much of the SE USA, Gulf of Mexico, and Caribbean Sea. Lionfish negatively impact fish and invertebrate assemblages and abundances, thus further spread is cause for concern. To date, the fish has not been reported on the Pacific coast of North or Central America. Here we examine the possibility of ballast water transfer of lionfish from colonized areas in the Atlantic Ocean to USA ports on the Pacific coast. Over an eight-year period, we documented 27 commercial vessel-trips in which ballast water was loaded in colonized sites and later discharged untreated into Pacific coast ports in the USA. California had the highest number of discharges including San Francisco Bay and Los Angeles-Long Beach. A species distribution model suggests that the probability of lionfish establishment is low for the western USA, Colombia and Panama, low to medium for Costa Rica, Nicaragua, El Salvador and Guatemala, medium to high for mainland Ecuador, and very high for western Mexico, Peru and the Galapagos Islands. Given the species’ intolerance of freshwater conditions, we propose that ballast water exchange be conducted in Gatún Lake, Panama for western-bound vessels carrying ‘risky’ ballast water to prevent invasion of the eastern Pacific Ocean.

## Introduction

Fishes have been introduced globally by vectors including sport-fish, food-fish and ornamental-fish industries, by human-created waterways, and by foreign ballast water carried and discharged by ships into novel environments. Ballast water is a proven method for species transfer. Wonham et al. [1] identified 32 fish species that were transported and introduced to a new location via ballast water, 24 of which established viable populations at the new site. Rahel [2] reported that five of 54 fish species introduced into the Nearctic region arrived via ships' ballast water.

Lionfish are native to the Indo-Pacific region, but have staged some of the world’s most spectacular and problematic invasions of global marine ecosystems, including the Caribbean Sea [3]. The species was first reported off the Florida coast in 1985, but later spread along the southeastern coast of the USA, throughout the Gulf of Mexico, and across the entirety of the Caribbean Sea including northern South America [4–6].

Introduced lionfish comprise two morphologically similar but genetically distinct species, the Red Lionfish *Pterois volitans* and the Devil Firefish *P*. *miles* (hereafter collectively referred to as lionfish) [7]. Both fishes are members of the family Scorpaenidae (scorpionfishes), two members of which have been reported in ballast water [1]. Johnston and Purkis [8] suggested that *P*. *miles* may have been transported into the Mediterranean Sea from the Suez Canal by ballast transfer, while Whitfield et al. [9] speculated that *P*. *volitans* could also be transferred by ballast water; however, Wonham et al.’s [1] survey identified neither lionfish species in ballast water.

Given their strong ecological impacts on fish and invertebrate communities in the Atlantic Ocean [10–12] there is growing concern that lionfish might replicate these effects if intentionally released [13] or accidentally discharged with ballast water [14] into the eastern Pacific Ocean. In this study we assess risk of dispersal and suitability of possible recipient areas in the eastern Pacific Ocean using shipping traffic data for the USA and a species distribution model (SDM), respectively.

## Methods

We utilized the National Ballast Information Clearinghouse (NBIC) database [15] for the years 2006 to 2013 inclusive to determine how many ships discharged and the number and volume of discharges of ‘risky’ ballast water that occurred into Pacific coast ports of the USA. Risky ballast water is defined as that which was loaded at a port in the eastern USA, Gulf of Mexico or Caribbean Sea—in an area where lionfish have been reported—following which the vessel crossed through the Panama Canal and discharged this water into a Pacific coast port without first conducting ballast water treatment or exchange. While the USA receives about 100,000 vessel arrivals per year, the number of vessels that conform to each of the aforementioned specific requirements is dramatically lower. Reports were submitted by ships' crews to the National Ballast Information Clearinghouse maintained by the Smithsonian Institution as vessels entered the USA. We utilized information from this database regarding ballast management, including coordinates of loading, exchange, and discharge.

Environmental suitability of recipient areas on the west coast of North America and Central America were modeled using an SDM; Hawaii was excluded from analysis. While SDMs have a number of built-in assumptions, they have proven useful in a large number of cases in identifying potentially inhabitable habitats [16,17]. As the statistical basis of the SDM, we used a General Additive Model (GAM), and the mcgv library [18] in R 3.3.0 [19]. GAMs have been shown to perform well and produce robust predictions, even for complex relationships [20].

The GAM model was built using environmental conditions to discriminate between presences and pseudoabsences at different locations in the invaded range in the Atlantic Ocean, using a binomial family and logit link function. We obtained environmental data provided by AquaMaps [21] at a scale of 0.5 degree latitude x 0.5 degree longitude cells worldwide. AquaMaps represents a valuable source as it provides environmental data for several dozen variables. All lionfish presence points on the east coast -spanning the years 1992 to 2015 (September)—were obtained from the U.S. Geological Survey [22], and matched to the nearest Aquamaps environmental cell, resulting in 383 unique presence cells. Lionfish occurrences and environmental data were not matched temporally. Instead, multi-year annual averages were used, as supplied and compiled from a variety of sources by AquaMaps.

We did not include data from lionfish’s native range, as key environmental characteristics (e.g. depth of occurrence) from this area are too conservative given lionfish occurrences in the invaded range. Evangelista et al. [23] noted that invaded range alone or the invaded range combined with native range provided better predictions of where lionfish occurred in the Western Atlantic Ocean than native range alone. Pseudoabsences were taken as all other cells within two-degree longitude of the eastern coastline of the Americas to match with distances of presence cells to the coastline (377/383 cells were within 2 degrees longitude). Given the relative roughness of the environmental cells, we did not need to randomly sample pseudoabsences, but instead included all cells not containing a lionfish occurrence point, resulting in 2640 unique pseudoabsence cells.

We considered 18 variables (area in the cell from 0–20m, 20–40m, 40–60m, 60–80m, and 80–100m in km^2,^, minimum, maximum and mean depth (m), minimum, max, mean, and range of sea surface temperatures (SST) (°C), minimum, max, mean of salinity (‰), sea bottom temperature (°C), salinity bottom mean (‰), and estuary area (km^2^)) of which 12 remained after removing highly collinear ones (r>0.8): five variables relating to areal size at different depths (area in the cell from 0–20m, 20–40m, 40–60m, 60–80m, and 80–100m in km^2^), minimum and mean depth (m), mean annual range of sea surface temperatures (SST)(°C), mean annual minimum SST (°C), mean annual sea bottom temperature (SBT)(°C), mean annual salinity (‰), and estuary area (km^2^). The initial subset of 18 variables mentioned above performed better and contained more biologically relevant variables in the final model than when we considered a larger complement of variables.

To determine the final model, we removed each variable one at a time, and compared the adjusted pseudo-r^2^ of the reduced model to the full model, and kept those variables that uniquely explained >1% of the variation. This is analogous to a Type III Sum of Squares procedure, except that we did not rely on statistical significance as our metric determining variable retention, because our use of pseudoabsences made the degrees of freedom questionable, and the effect size (r^2^) was arguably a more meaningful metric of fit (and was more conservative).

**Fig 1:**
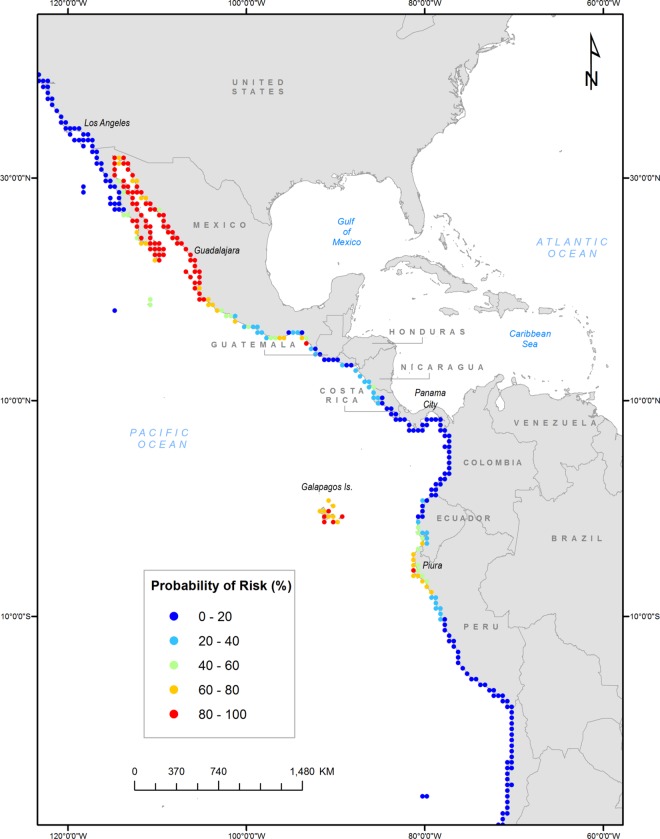
Predicted suitability for lionfish on the west coast of North, Central and South America. Made with Natural Earth.

We used the resulting variable list to create the final SDM, and present both the final pseudo r^2^_adj_ as well as the Area Under the Curve (AUC) value of a Receiver Operating Curve (ROC) as a common measure of discrimination between presences and absences, using the pROC library in R [24]. Then, we conducted a sensitivity analysis of establishment probabilities associated with each variable in the final model. For each variable in turn, we increased the value incrementally from the minimum to the maximum observed across the east coast, holding all other variables at the mean value where lionfish occur; therefore, these values should reflect permissive conditions. This analysis provided information on which variable was critical to a high probability of establishment. We applied the SDM to the west coast of the Americas to estimate probability of establishment, given sufficient propagules. Finally, a geographic information system was utilized to map the spatial distribution of model results. Five probability classifications were established and plotted using an equal interval approach (Fig 1).

## Results

United States ballast water records indicated a total of 27 vessel-trips between 2006 through 2013 in which ballast water was loaded in ports known to be inhabited by lionfish, with subsequent passage of those vessels through the Panama Canal and discharge of untreated ballast water into western USA ports. The top recipient area was San Francisco Bay, California, with three vessels discharging into each of Oakland, Richmond, and San Francisco plus one into Benicia (Table 1). The combined ports of Los Angeles—Long Beach, California were next highest, with eight vessels discharging risky ballast water, followed by Portland, Oregon with three (Table 1). Six other areas in California, Oregon, or Washington each received single ships that discharged eastern-sourced ballast water. Los Angeles received the largest number of ballast tank discharges (26), followed by Oakland, Richmond, and Portland (Table 1). However, the largest volumetric discharges occurred into Los Angeles and San Francisco (Table 1).

**Table 1:** Ports in the western USA (exclusive of Hawaii) that received discharges of untreated ballast water that was sourced from the western Atlantic Ocean, Caribbean Sea or Gulf of Mexico where lionfish are known to occur.

Location	Number of Vessel Trips	Volume Discharged (m^3^)	Number of Tanks Discharged
Los Angeles/Long Beach, CA	8	24096	29
Portland, OR	3	3094	5
San Francisco, CA	3	11785	4
Richmond, CA	3	4141	5
Oakland, CA	3	1250	6
Longview, WA	1	706	1
Astoria, OR	1	77	1
Tacoma, WA	1	710	3
Everett, WA	1	182	2
Benicia, CA	1	1366	4
San Diego, CA	1	142	1
Seattle, WA	1	147	2

**Fig 2:**
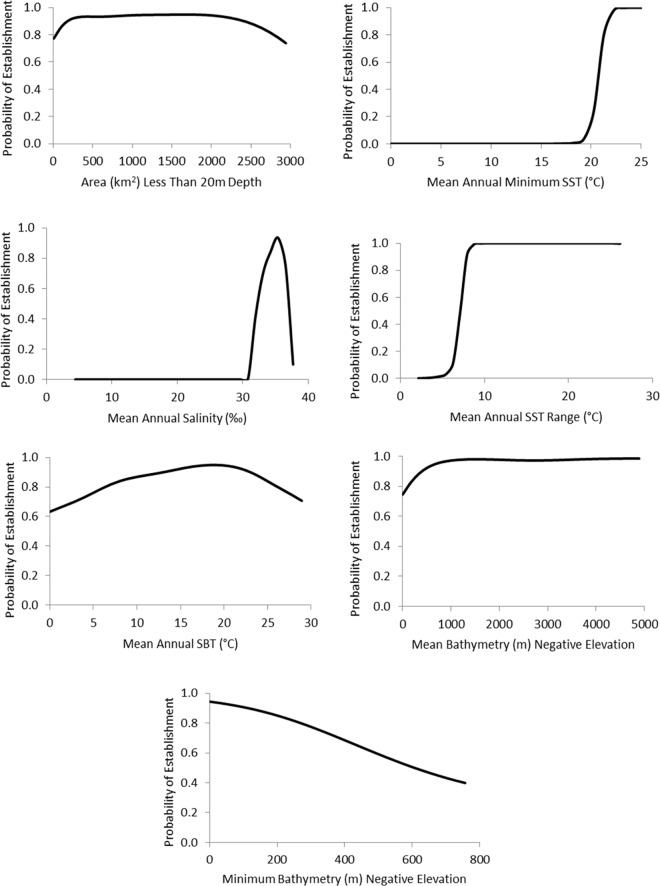
Relationship between predicted establishment probability and key environmental parameters.

We found that seven variables—areas less than 20m depth, minimum depth, mean depth, mean annual salinity and mean annual minimum SST and mean annual Sea Bottom Temperature (SBT), as well as mean annual range of SST- all explained at least 1% of unique variation (Table 2). In combination, the final SDM explained 46% of the variation (pseudo-r^2^_adj_ = 0.456), and yielded an AUC value of 0.95 indicating strong discriminatory power. For our sensitivity analysis, we started with the mean value of observed lionfish occurrences for each environmental variable in the final SDM. At these mean values, the probability of establishment was 0.53. Modifying each variable in isolation suggested that, within the range occurring on the east coast, mean annual minimum sea surface temperature, mean annual range of SST and mean salinity were critical to establishment (Fig 2). Amount of area less than 20m, minimum and mean depth and mean annual sea bottom temperature had much smoother, less pronounced relations with probability of establishment across the range of values observed on the east coast (Fig 2).

**Table 2:** Factors retained and their weightings in species distribution model to determine potential range of lionfish if introduced to the west coast.

Variable	Unique Variance Explained
Area (km^2^) less than 20m depth	0.028
Minimum bathymetry (m) negative elevation	0.022
Mean bathymetry (m) negative elevation	0.012
Mean annual SST range (°C)	0.093
Mean annual minimum SST (°C)	0.078
Mean annual SBT (°C)	0.010
Mean annual salinity (‰)	0.034

Application of the final SDM to the west coast produced the full range of probabilities of establishment, from zero to 0.988, with the highest risks in Mexico, Peru and the Galapagos Islands (Fig 1). Other high risk regions include Peru and Ecuador (Fig 1). In western Panama, risk of establishment ranged from a low of 0.018 to a high of 0.11, with a mean of 0.04. Probability of establishment was >0.1 for 19.4% of cells, >0.5 for 8.0% of cells, and >0.8 for 7.5% of cells. Probability of establishment was low for all locations in the western USA, including contiguous states plus Alaska (Fig 1).

## Discussion

Our study revealed that a number of ports in the western United States received untreated ballast water that was sourced from ports where lionfish have been reported. Los Angeles received the greatest number of ship trips in which Atlantic Ocean ballast was discharged, the largest number of tanks emptied, and the largest volume of ‘risky’ ballast water. Its sister port of Long Beach had additional discharges. The other principal discharge region where discharges occurred on the west coast was San Francisco Bay, encompassing the ports of Oakland, San Francisco, Richmond and Benicia. The most southern port, San Diego, had only a single vessel that discharged a relatively low-volume ballast tank (142 m^3^). Portland, Oregon is located on the Columbia River, and thus would be non-vulnerable to colonization even if it received high propagule pressure.

Ballast water is drawn in through a sea chest, which has a screen at the entrance to prevent large individuals or debris from entering ballast tanks. Routine pore size on these screens (holes 15–25mm diameter or slots 20–35mm width [25]) can equal or exceed the size of many larval fishes, including lionfish whose reported larval stages range from 1.5 to 11mm [26–28]. Cross-sectional area of these fishes is the more relevant metric and would be even smaller. Most fishes recovered in ballast tanks are juveniles or are otherwise small individuals [1].

Owing to their small size, we anticipate that larval lionfish could be drawn into sea chests without injury when taken up prior to settlement, which occurs between 20 and 35 days after hatching [29]. These individuals could easily fit through sea chests and their associated coarse-sized grates. For example, a 15–16 day old larval individual is ~ 8mm standard length [28], and at settlement is between 10 and 12mm [30]. Furthermore, given that lionfish can reproduce every 2–4 days and produce up to 25,000 eggs per reproductive bout or approximately two million eggs per year [31,32], it seems likely that larval lionfish will be taken up through sea chests at some frequency. In addition, sea chests without proper maintenance are likely to have even larger sized holes in their intake grating, allowing larger individuals to be uploaded. For example, the grates on some operational vessels are lost or compromised [Ruiz, pers. obs.], and fish as large as 39.2 cm been found in ballast tanks [1]. The extent to which lionfish can survive entry into the tank and during transit in ballast water until the time of discharge are essential but unanswered questions.

Being introduced to a site is the second of three prerequisites for successful establishment by a nonindigenous species [1]. Species must then establish a successfully reproducing population. Johnston and Purkis [14] modeled areas in the eastern Pacific Ocean susceptible to an accumulation of lionfish individuals sufficient to favour establishment, and suggested that risk from ballast discharge is highly patchy and limited mainly to Panama and Puerto Vallarta, Mexico, with California seemingly at lower risk of population establishment. Even though these authors argued that subsequent dispersal was unlikely due to low natural connectivity between sites, coastal movement of vessels carrying ballast water could facilitate secondary spread. While we lack ballast water records for Mexico and Panama, our SDM also suggests that much of western Mexico is highly suitable for establishment of lionfish, while Panama and California are not currently at risk (Fig 1). Models developed by Evangelista et al. [23] that used only non-native distributions of lionfish in the training dataset also suggested that regions of Western Mexico and Guatemala were suitable habitat for lionfish. Furthermore, FishBase Information and Research Group (FIN) [33] developed an SDM for *P*. *volitans* that predicted suitable habitat based on salinity, temperature, depth and other features. This model also predicted that lionfish will not survive in west coast American ports but could do very well at locations in Central America including northern Mexico. Finally, Poursanidis [34] modeled possible distribution of *P*. *miles* and identified only the southern tip of California would provide any measure of suitable environment, though Central America had a patchy but often higher risk.

Based upon our and other models [14,23,33,34] it would appear that even if lionfish were introduced to west coast ports in the USA, they are unlikely to survive. However, the predicted distribution as defined by all aforementioned models is constrained by the lack of a complete dataset—particularly for nearshore environments including bays and estuaries—as lionfish are still expanding their range, including to colder, lower salinity, and deeper waters. If thermal or salinity thresholds are lower than those used by these models—as is suggested by lab studies and by anecdotal observation—certain parts of the country, notably the region from San Diego to Los Angeles, could be at risk. For example, the mapping parameters [33] used as absolute minimum and preferred minimum sea surface temperatures for *P*. *volitans* were 18°C and 21.85°C, respectively. However, lionfish have been observed in their invaded range at much lower water temperatures (i.e. 13.8°C; [35]). Furthermore, lab studies have found that this species can acclimate to 12.5°C (Wayne Bennett, pers. comm.), has an absolute critical minimum of 9.5°C (mean 12.67°C) [36], and a mean chronic lethal minimum of 10.0°C [37]. It should be noted that both Barker [36] and Kimball et al. [37] used juvenile lionfish in their experiments, which is particularly relevant since this life stage is most likely to be transferred in ballast water.

The FIN [33] model used an absolute minimum surface salinity of 31.55‰. However, Jud et al. [38] observed that fish held at 7‰ were no different with respect to mortality, behavior and growth to those held at 35‰ over a 28-day period, though these results should be interpreted with caution since replication was low. However, these findings are consistent with observations of Jud et al. [39], who identified lionfish in low salinity and temperature regions of the Loxahatchee River. In addition, Schofield et al. [40] found that lionfish survived exposure to a salinity of 5‰ for a maximum of 12 days before dying and were able to survive for the duration of the experiment (32 days) at a salinity of 10‰ while continuing to feed and grow.

Thermal and salinity tolerance data for larval lionfish are largely lacking, and dependence of species distributions models based on presence data for other life stages can therefore be risky. It is imperative that research on tolerances be conducted for different life stages to ensure that species distributions models reflect likely scenarios. In addition, further exploration of depth limits is warranted. Although our model suggested a negative relationship between minimum depth and establishment success, the pattern is reversed for mean depth (Table 2). Lionfish have been observed at depths down to 300m even though the species’ depth limits are not well established [28,41,42]. Remotely operated vehicle surveys may help clarify depth limits of the species [43]. Lionfish presence is determined by a combination of temperature and depth. Individuals can be found at disproportionately greater depths in certain areas depending on regional temperature regimes as defined by oceanic bottom structure [37,42]. This interplay between temperature and depth may lead to erroneous conclusions that lionfish are restricted by depth when, in fact, the limiting variable is temperature. Biases by recreational dive reports further enforce this misconception [14].

Even though California appears to be environmentally inhospitable to survival of lionfish, we propose that discharges of untreated ballast water from Atlantic ports be considered risky until proven otherwise given uncertainties in models currently available. Some regions of Central and South America identified in our and other models appear to be highly suitable for establishment of lionfish, though lack of data pertaining to potential introduction effort (i.e. untreated ballast water discharges of Atlantic Ocean water) compromises our ability to assess risk with greater confidence.

Our SDM identified the Galapagos Islands as highly suitable for lionfish establishment. Keith et al. [44] previously identified lionfish as a potential invasive species for the Galapagos Marine Reserve. Given the past history of marine invasions in the region and increased tourism and commercial ship traffic [44,45], increased vigilance is necessary to prevent introduction of either species to these islands. However, commercial traffic to the Galapagos Islands from outside Ecuador is not currently permitted, reducing risk of ship-mediated invasions.

Our modeling effort has a number of limitations. Our model used lionfish occurrence and environmental parameter data from different years. If a time trend existed with respect to key environmental parameters highlighted by our models, it is possible that one could erroneously ascribe higher or lower importance to that variable. Our model can be refined for areas where lionfish might be expected to occur by utilization of datasets (i.e. HYCOM) that provide higher spatial and/or temporal resolution. These datasets could inform predictions secondary spread if lionfish were to colonize a given region.

Prevention of lionfish introduction to the west coast of North and Central America should be a management priority given the pattern of spread and strong ecological impacts of these species in the western Atlantic Ocean. One prong of this approach ought to include mandatory ballast treatment or exchange for the relatively small number of vessels that carry ballast water from Atlantic to Pacific coasts via the Panama Canal, exchanging saline water in ballast tanks for freshwater from Gatún Lake. Vessels require up to 12 hours to transit the canal [46], which may be insufficient time to complete ballast water exchange. In that case, a vessel would have to lay up in the lake for a short period during which ballast exchange is conducted. It is not possible to lay up on the Atlantic approach to the canal system, as water salinity is too high (14.4–24.5‰; [47]) in Limón Bay to kill lionfish. Lionfish can survive prolonged exposure to 10‰ salinity [40] and for up to 94 hours at 4‰ [38]. Considering that the canal is within and controlled by Panama, its consent would be required for any ballast discharges. The volume of salt water discharged into the lake by vessels would be miniscule considering the very small number of vessels that would be affected. Although it is controversial to recommend ballast water release into a water body utilized as a drinking water source by Panamanians, marine organisms are unlikely to survive in the freshwater environment. Exchanged saltwater-ballast discharges are permitted—and mandated unless otherwise treated—into the Great Lakes, which are also a principal drinking water source for many municipalities. In both cases, such a policy may serve to reduce risk of biological invasions.

## Conclusions

Lionfish have quickly spread across the SE Atlantic coastline of the USA [4,5,9], with strong ecological effects [10–12]. We forecasted possible spread to the western USA via ballast water transfer in vessels using the Panama Canal. Twenty-seven vessels were identified that discharged ‘risky’ ballast water to ports in California, Oregon and Washington state. A Species Distribution Model revealed that current conditions in these ports would likely be inhospitable to lionfish establishment, though other countries in Central and South America would be highly suitable.
